# 

**Published:** 1895-05-25

**Authors:** 


**Notices of Births, Deaths, and Marriages** are inserted in this column at a charge of 2s. 6d. for an announcement not exceeding four lines.

**Notice to Advertisers and Subscribers.**

All Advertisements, Orders for Copies of The Hospital and Business Communications should be addressed to **The Manager, NOT TO THE EDITOR,** The Hospital, 428, Strand, London, W.C.

The Cost of Subscription, post free, is as follows:—Per week, 2½d.; per quarter, 2s. 8d.; per half-year, 5s. 3d.; per annum, 10s. 6d. Foreign:—Per Annum, 15s. 2d.; payable, in all cases, in advance.

### NOTICE TO CORRESPONDENTS.

All MS., Letters, Books for Review, and other matters intended for the Editor should be addressed The Editor, The Lodge, Porchester Square, London, W.

The Editor cannot undertake to return rejected MS., even when accompanied by stamped directed envelope.

**"Burdett's Hospital Annual," 1895,**

Ready Immediately.

Sixth year of publication. About 1000 pp. Scarlet cloth, gilt lettered. Price 5s. A most complete guide to British, Colonial, Indian, and American Hospitals, Dispensaries, Nursing and Convalescent Institutions, and Asylums. A few copies of the "Annual" for 1894 may still be obtained on application to the Publishers, The Scientific Press (Limited), 428, Strand, London, W.C.

**NOTICE.—Vol. XVII. of "The Hospital" (October, 1894, to April, 1895), in handsome cloth binding, is now on sale at the Office of this Journal. Price 6s. Cases for binding the Numbers contained in this Volume, 1s. 6d. Index to Vol. XVII., 2½d., post free.**

### THE HOSPITAL FOR SICK CHILDREN.

### SCRAPS AND GLEANINGS.

A bacteriological institution is about to be erected at Leicester. The local medical officer of health will direct the same.

The Leper Hospital of St. Stephen's, in the County of Waterford (Ireland) is to be converted into a county infirmary.

Donations to the extent of £2,131 are announced to have been received at the festival dinner of the Hospital for Sick Children, Great Ormond Street.

The report of the Royal Commission on tuberculosis, to which we have previously referred, is now effected, and was arranged to be definitely presented to Parliament last week.

"Physicienne" has been suggested by a weekly contemporary as a title for female members of the medical profession as being a more euphonious designation than lady doctor.

At the opening of the St. Mary's Hospital bazaar at Portman Rooms on June 27th by the Princess of Wales, she will be conducted through the bazaar by the Duke of York as president of the hospital.

The date of the opening of the bazaar in aid of the Poor Sick Children's Seaside Holiday Fund by the Countess Cadogan at the Queen's Gate Hall has been altered from the 27th to the 26th June.

There is an actual decrease of £200 on the voluntary contributions towards the Bath Mineral Waters Hospital this year. Considering the very large number of London poor sent here annually, the committee comment on the fact that the Metroplitan Hospital Sunday Fund have not awarded any contribution this year to their institution.

### Books Received.

Iliffe and Son.

"Holiday Tramps Through Picturesque England and Wales."

J. and A. Churchill.

"Elements of Health." Louis Parkes, M.D.

"Mentally Feeble Children." Fletcher Beach.

Kegan Paul.

"Bradshaw's Bathing Places and Health Resorts."

H. K. Lewis.

"Manual of Gynæcological Practice." Taylor and Edge.

"Surgical Diseases of Children." D'Arcy Power.

**Periodicals and Pamphlets.**—Science, Monthly Homœopathic Review, The Tri-State Medical Journal, Invention, American Journal of Insanity, The Art Bible (Part II.), Strand Magazine, Strand Musical Magazine, The Picture Magazine, Round the Coast (Part I.), The Child's Guardian, Electrical Review, Industries and Iron, The Planter, Guy's Hospital Gazette, The Zenana, The Sun, Westminster Review, Humanitarian, Cassell's Magazine, The Spastic and Tabetic Types of General Paralysis, Penny Tales for the People, The English Illustrated, The Corpuscle, Leisure Hour, Sunday at Home, Girl's Own Paper, Boy's Own Paper, The Therapist.

### The Student of Medicine.

### APPOINTMENTS.

A. W. W. Baker, M.D.Dubl., F.R.C.S.I., appointed Examiner in Dental Surgery and Pathology at the Royal College of Surgeons in Ireland. F. Barnes, M.D., appointed Consulting Physician to the British Lying-in Hospital. J. Barton, M.D., F.R.C.S.I., appointed Examiner in Anatomy at the Royal College of Surgeons in Ireland. A. W. Bate, M.D.St. And., F.R.C.S.I., appointed Examiner in Midwifery and Gynæcology at the Royal College of Surgeons in Ireland. J. J. Burgess, L.R.C.P., F.R.C.S.I., appointed Examiner in Pathology at the Royal College of Surgeons in Ireland. A. Chance, L.R.C.P., F.R.C.S.I., appointed Examiner in Surgery at the Royal College of Surgeons in Ireland. A. J. Collis, M.A., M.B., B.C.Cantab, M.R.C.S., L.R.C.P., appointed Resident Medical Officer at the Weston-super-Mare Hospital and Dispensary. Dr. Conway, appointed Deputy Medical Officer to the Workhouse of the Uxbridge Union. C. Coppinger, M.D., F.R.C.S.I., appointed Examiner in Physiology and Histology at the Royal College of Surgeons in Ireland. H. C. Ensor, M.R.C.S.Eng., L.S.A., appointed Honorary Ophthalmic Surgeon to the Newport and Monmouthshire Infirmary. F. W. Fish, M.B. Ch.B., appointed Resident Medical Officer to the Southport Convalescent Hospital and Sea Bathing Infirmary. E. C. Garland, L.R.C.P.Edin., M.R.C.S.Eng., reappointed Medical Officer of Health to the Yeovil Town Council. L. N. Harding, B.A., M.B., M.R.C.S., L.R.C.P.Lond., appointed House Surgeon to the Brighton, Hove, and Sussex Throat and Ear Hospital. F. Haydon, L.R.C.P.Lond., appointed Secretary to the Court of Examiners of the Society of Apothecaries. B. C. Kendall, M.R.C.S., L.R.C.P., appointed Medical Officer to the Helston Dispensary. E. O. Kingdon, L.R.C.P.Lond., M.R.C.S.Eng., reappointed Medical Officer for the No. 3 District of the Holdsworthy Union. J. Knott, M.D., F.R.C.S.I., appointed Examiner in Chemistry and Physics at the Royal College of Surgeons in Ireland. E. Lapper, F.R.C.P., L.R.C.S.I., appointed Examiner in Chemistry and Physics at the Royal College of Surgeons in Ireland. C. H. Leaf, M.A., M.B., B.C.Cantab., M.R.C.S., appointed Assistant Demonstrator of Anatomy at the London Hospital. W. McClelland, M.B., Ch.B.Vict., reappointed Non-Resident Medical Officer to the Gynæcological Department of the Liverpool Royal Infirmary. W. A. Mackay, M.B., C.M.Glas., appointed Assistant Medical Officer to the City of Glasgow Parochial Board. P. W. Maxwell, M.D.Edin., F.R.C.S.I., appointed Examiner in Ophthalmology at the Royal College of Surgeons in Ireland. T. Myles, M.D., F.R.C.S.I., appointed Examiner in Anatomy at the Royal College of Surgeons in Ireland. G. H. Oliver, M.R.C.S.Eng., L.R.C.P.Lond., L.S.A., appointed Assistant Surgeon to the Bradford Eye and Ear Hospital. R. J. Paton, M.B., C.M.Edin., appointed Honorary Surgeon to the In-patients at the Newport and Monmouthshire Infirmary. R. G. Patteson, M.B., F.R.C.S.I., appointed Examiner in Pathology at the Royal College of Surgeons in Ireland. J. D. Pratt, M.D.Dubl., F.R.C.S.I., appointed Examiner in Medical Jurisprudence at the Royal College of Surgeons in Ireland. A. G. Robb, M.B.R.U.I., B.Ch., appointed Medical Officer for No. 10 Sub-district (Springfield) of the Belfast Dispensary. Dr. Roll, appointed Honorary Assistant Physician to the Leicester Infirmary. L. Roper, M.A., M.B., B.C.Cantab., reappointed Clinical Assistant and Demonstrator in the Throat Department at Guy's Hospital. J. A. Scott, M.D., F.R.C.S.I., appointed Examiner in Physiology, Histology, and Biology at the

THE STUDENT OF MEDICINE—*continued.*

Royal College of Surgeons in Ireland. R. T. Stack, M.D.Dubl., F.R.C.S.I., appointed Examiner in Dental Surgery and Pathology at the Royal College of Surgeons in Ireland. Dr. W. E. Stanton, appointed Medical Officer for the Deeping St. Nicholas District of the Spalding Union. Sir W. Stokes, M.D., F.R.C.S.I., appointed Examiner in Surgery at the Royal College of Surgeons in Ireland. J. B. Story, M.B.Dubl., F.R.C.S.I., appointed Examiner in Ophthalmology at the Royal College of Surgeons in Ireland. Dr. J. C. Taylor, appointed Medical Officer for the Third District of the Westbury and Whorwellsdon Union. Dr. Weston, appointed Medical Officer for the Leighford District of the Stafford Union. G. B. White, M.B.Dubl., F.R.C.S.I., appointed Examiner in Biology at the Royal College of Surgeons in Ireland. T. M. Wills, F.R.C.S.I., Senior Honorary Surgeon, appointed Consulting Surgeon to the Bootle Borough Hospital, Liverpool.

### VACANCIES.

The following vacancies are announced this week, the amount of salary in each case being given after the name of the institution:

### Resident Medical Officers.

Addenbrooke's Hospital, Cambridge.—Resident House Physician. Salary £65 per annum, with board, lodging, and washing in the hospital. Applications to the Secretary by May 30th.

Cardiff Infirmary.—Senior Resident Medical Officer, doubly qualified. Salary £100 per annum, with board, washing, and furnished apartments. Applications to George T. Coleman, Secretary, by May 27th.

General Hospital, Birmingham.—Assistant House Surgeon. Must possess surgical qualification and be registered. Appointment for six months. No salary, but residence, board, and washing provided. Applications, with certificate of registration and copies of testimonials, to H. J. Collins, House Governor, by June 1st.

Guardians of Kensington—Second Assistant Resident Medical Officer for the Workhouse and Infirmary. Age between 21 and 30 years. Doubly qualified. Salary £80 per annum, with apartments, board, and washing. Applications on forms to be obtained at the office of the Clerk to the Guardians, Marloes Road, Kensington, where they must be delivered by ten a.m. on May 25th.

Liverpool Stanley Hospital.—Junior House Surgeon. Must possess a registered medical and surgical qualified qualification. One who has filled a similar office preferred. Salary £70 per annum, with board, &c. Applications and testimonials by May 30th.

North-Eastern Hospital for Children, Hackney Road, Shoreditch, N.E. —House Physician. Appointment for six months, and at the expiration of this time the House Physician will be required, if eligible, to serve as House Surgeon for a further period of six months. Must possess a medical and surgical qualification. Salary as House Physician at the rate of £60 per annum, and as House Surgeon at the rate of £80 per annum. Applications and testimonials to T. Glenton-Kerr, Secretary, City Office, 27, Clement's Lane, Lombard Street, E.C., by June 11th.

New Hospital for Women, 144, Euston Road, N.W.—Two qualified Medical Women as House Surgeons. Applications to the Secretary by May 29th.

Parish of Birmingham.—Resident Medical Officer for the Workhouse. Doubly qualified and registered. Salary £150 for the first year, rising £10 yearly to £200 per annum, rations, apartments, washing, and attendance. Applications to be made on printed form, to be obtained from Walter Bowen, Clerk to the Guardians, Parish Offices, Edmund Street, to be returned to him by May 25th.

Rotherham Hospital and Dispensary.—Resident House Surgeon; doubly qualified. Salary £100 per annum, with rooms, washing, and commons. Appointment for three years. Applications to the Secretary by the end of May.

Royal Free Hospital, Gray's Inn Road, W.C.—Two Resident Medical Officers. Doubly qualified. Appointment for six months, but the holder will be eligible for re-election. No salary, but board, residence, and washing. Applications and testimonials to Conrad W. Thies, Secretary, by June 1st.

### Non-Resident Medical Officers.

Dental Hospital of London, Leicester Square.—Two Dental Surgeons. Candidates must be Licentiates of Dental Surgery of one of the licensing bodies recognised by the General Medical Council. Applications and testimonials to J. Francis Pink, Secretary, by June 10th.

Great Northern Central Hospital, Holloway Road, N.—Pathologist and Registrar. Appointment for one year. Honorarium at the rate of fifty guineas per annum. Applications to the Secretary by May 27th.

Holborn District.—Medical Officer of Health. Salary £350 per annum. Applications on printed forms to be obtained from Matthew H. Hale, Clerk to the Board, Holborn Town Hall, accompanied by not more than three testimonials, to be returned to him before May 30th.

Royal College of Surgeons of England.—Hunterian Professors, the Erasmus Wilson Lecturer, and the Arris and Gale Lecturer for the ensuing year. Applications, with particulars of subjects upon which it is proposed to lecture, to E. Trimmer, Secretary, by June 6th.

Royal Hospital for Women and Children, Waterloo Bridge Road, E.C.—Clinical Assistant and Anæsthetist required for six months. Doubly qualified. Salary at the rate of £30 per annum. Applications and testimonials to R. Gerrard Kestin, Secretary, by May 29th.

Royal Westminster Ophthalmic Hospital, King William Street, West Strand.—Clinical Assistants. Appointments for six months. Must be duly qualified, and preference will be given to those who have had previous experience of ophthalmic practice. Applications and testimonials to T. Beattie-Campbell, Secretary, by June 1st.

St. Mary's Hospital Medical School.—Lecturer on Mental Diseases Applications to G. P. Field, Dean, by May 27th.

St. Thomas's Hospital Medical School.—Lecturer on Physiology. Applications to the Dean by May 25th.

Society of Apothecaries.—Examiner in Medicine (including Forensic Medicine, Pathology, and Midwifery). Applications to J. R. Upton, Clerk to the Society, Apothecaries' Hall, Blackfriars, by May 27th.

**Medical Appointment.**

NORTH-EASTERN HOSPITAL for CHILDREN,
HACKNEY ROAD, SHOREDITCH, N.E.

T. GLENTON-KERR, Secretary.

